# Ion uptake in naturally acidic water

**DOI:** 10.1007/s00360-024-01552-6

**Published:** 2024-04-23

**Authors:** R. J. Gonzalez, M. L. Patrick, A. L. Val

**Affiliations:** 1 Department of Biology, University of San Diego, 5998 Alcalá Park, San Diego, CA 92110 USA; 2 Laboratório de Ecofisiologia E Evolução Molecular, Instituto Nacional de Pesquisas da Amazônia (INPA), Manaus, Amazonas Brasil

**Keywords:** Ion transport, Characiformes, Zebrafish, Acidic waters

## Abstract

The first studies on ion regulation in fish exposed to low pH, which were inspired by the Acid Rain environmental crisis, seemed to indicate that ion transport at the gills was completely and irreversibly inhibited at pH 4.0–4.5 and below. However, work on characid fish native to the Rio Negro, a naturally acidic, blackwater tributary of the Amazon River, found that they possess ion transport mechanisms that are completely insensitive to pHs as low as 3.25. As more species were examined it appeared that pH-insensitive transport was a trait shared by many, if not most, species in the Order Characiformes. Subsequently, a few other species of fish have been shown to be able to transport ions at low pH, in particular zebrafish (*Danio rerio*), which show rapid recovery of Na^+^ uptake at pH 4.0 after initial inhibition. Measurements of rates of Na^+^ transport during exposure to pharmacological agents that inhibit various transport proteins suggested that characiform fish do not utilize the generally accepted mechanisms for Na^+^ transport that rely on some form of H^+^ extrusion. Examination of zebrafish transport at low pH suggest the rapid recovery may be due to a novel Na^+^/K^+^ exchanger, but after longer term exposure they may rely on a coupling of Na^+^/H^+^ exchangers and NH_3_ excretion. Further work is needed to clarify these mechanisms of transport and to find other acid-tolerant species to fully gain an appreciation of the diversity of physiological mechansisms involved.

## Introduction

In the 1970’s it was recognized that acidified rainfall was impacting lakes and streams throughout the northeastern United States and parts of Europe and killing fish fauna inhabiting those waters. Much research into the causes ensued, and by the early 1980’s it was clearly shown that waters with pH values below 5.0 were lethal to many fish species and that the primary cause of death was disruption of ion regulation (see review by McDonald 1983). Acidified waters inhibited active Na^+^ and Cl^−^ uptake and at around pH 4.0–4.5 inhibition was total (McDonald and Wood 1981). At the same time, low pH greatly stimulated diffusive ion losses. Together these effects produce a net loss of ions, which causes plasma ion concentrations to plummet. This in turn, initiates internal osmotic disturbances that culminate in cardiovascular failure and death when fish lose 30–50% of their whole-body Na^+^ and Cl^−^ (Packer and Dunson 1970, 1972; Milligan and Wood 1982).

Not long after research on the effects of acid rain began it was recognized that there were naturally occurring freshwater habitats on almost every continent that were just as acidic or even more acidic than the waters impacted by acid rain, and these waters were inhabited by a variety of fish species. Such observations raised the obvious question, how do these fish avoid disruption of ion balance and survive in these acidic environments? The earliest studies on fish species from North America, such as banded sunfish (*Enneacanthus obesus*), that inhabit naturally acidic swamps and bogs along the Atlantic Coastal Plain, showed that ion transport was just as sensitive to low pH as in sensitive species, and that the key to survival at low pH was an ability to resist stimulation of diffusive salt loss (Gonzalez and Dunson 1987, 1989). This basic response was seen in a variety of other species that were examined (Freda and McDonald 1988; Kwong et al. 2014). However, about a decade later when attention turned to fish species native to the Rio Negro, an ion-poor, acidic, black water tributary of the Amazon River, something very different was observed. An examination of Na^+^ transport in neon tetras (*Paracheirodon innesi*), a member of the species-rich tetra family Characidae, found that, unlike all prior species studied, Na^+^ uptake was completely unaffected by transfer to low pH water (down to pH 3.25; Gonzalez and Preest 1999). A second study showed Cl^−^ uptake in neon tetras was equally insensitive to low pH (Preest et al. 2005). Subsequent examination of other characids indicate that these specializations are widespread in the family (Gonzalez et al. 2002), and even extend to species outside the family Characidae, but within the order Characiformes (Gonzalez et al. 2017). There is evidence now that at least a few other fish from unrelated groups also possess ion transport mechanisms with some degree of low pH insensitivity, including a Rio Negro catfish (*Pimelodes* sp; Gonzalez et al. 2002), North American yellow perch (*Perca flavescens*; Freda and McDonald 1988), and zebrafish (*Danio rerio*; Clifford et al. 2021) which are native to India. That fishes from such diverse origins are able to transport salts at low pH suggests the trait may be more common than was first thought.

The purpose of this review is the summarize what is known about ion uptake in these species. We will briefly describe the mechanisms of ion transport generally used by freshwater fish and summarize how high H^+^ concentrations are thought to inhibit them. We will then review the functional and molecular/immunohistochemical evidence currently available for the mechanisms employed by acid-tolerant species. Before describing the physiology, however, we will start by briefly describing naturally acidic habitats. It is our hope that these descriptions will spur further research and uncover additional groups of acid-tolerant fish.

## Naturally occurring acidic waters

**Fig. 1:**
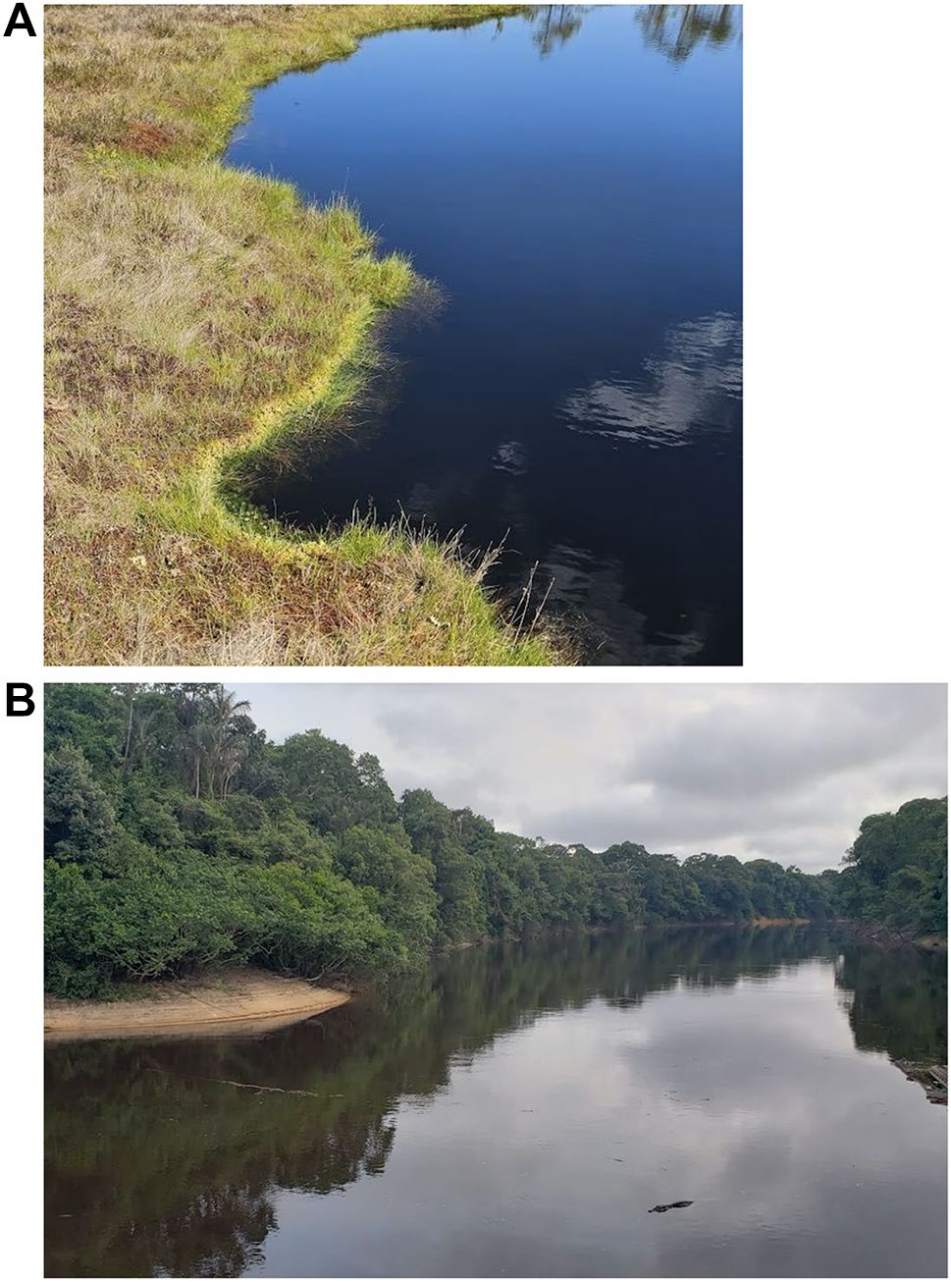
Peat bog at Sooma National Park, Estonia (**A**) and blackwater tributary of Rio Negro, Anavilhanas archipelago, Amazonas, Brazil (**B**). Photos: AL Val

Acidic bodies of water occur naturally all over the world due to geological conditions, organic activity, and atmospheric influences. In the simplest case, boreal streams can show a significant decrease in surface water pH during melting due to the natural dissociation of organic acids, as shown by Hruska et al. (2001). In addition, there are many examples of bodies of water that are acidic year-round. Volcanic lakes, such as Kawah Lakes in Indonesia, Boiling Lake in Dominica, Acid Lake in the crater of the Pinatubo volcano in the Philippines, and Lake Osorezan on Honshu Island in Japan, can be very acidic due to high levels of sulfates, and other compounds released from the underlying volcanic vent. Peat bogs, found mostly at higher latitudes in the northern hemisphere, can be quite acidic as a result of the accumulated peat *i.e.* a deposit of organic material, mainly from plants (Kuttim et al. 2018) that releases organic acids into the small bodies of water, making them acidic, as is the case in Soomaa National Park and other formations in Estonia (Triisberg et al. 2013) (Fig. 1A). Perhaps most common are black water river systems, which can be found along the Atlantic coastal plain of North America from Florida to New Jersey, the Tasik Bera in Malaysia, the Congo River system in Africa, certain streams in Queensland, Australia, and the Rio Negro sub-basin in Amazonia (Fig. 1B). This last river system is possibly the most well-known so let us take a closer look at it.

The Rio Negro drains an area of almost 700,000 km^2^ in the northwestern region of the Amazon basin. The soils are made up largely of silicate sands, which bind nutrients very loosely and as a result, the waters have exceptionally low concentrations of ions that vary between the two main seasons of the year in the Amazon, dry and wet. (see Table 1, compiled from data by Holland et al. 2017). A consequence of the low ion levels is a reduced bacterial diversity, which means that plant materials entering the river system are broken down very slowly. The resulting large quantities of natural organic matter (NOM), contain organic acids, primarily humic and fulvic acids (Leenheer 1980), acidify the river and produce the characteristic tea color in the water (and explain why it is called black water).

**Table 1:** Ionic composition and levels and characteristics of dissolved organic carbon (DOC) of the Rio Negro in the wet and dry seasons (data from Holland et al. 2017)

Parameter	Wet season (mg L^−1^)	Dry season (mg L^−1^)
Sodium	1.33	0.78
Calcium	0.16	0.17
Magnesium	0.11	0.08
Potassium	0.34	0.61
Chloride	0.74	0.5
Alkalinity (CaCO_3_)	12.4	7.7
DOC	8.75	5.84
SAC_340_ (cm^2^ mg^−1^)^a^	66.65	43.69
Fluorescence index^b^	1.39	1.41
SUVA_254_^c^	7.35	4.98
Abs_254/365_^d^	3.43	3.63
%Tyrosine-like	1.88	1.07
%Tryptophan-like	1.83	0.52
%Fulvic-like^e^	36.32	34.16
%Humic-like^e^	59.97	64.25

^a^SAC340, which is (cm^2^ mg^−1^). (2.303 × absorbance at 340 nm)/DOC, gives an indication of aromaticity of DOC

^b^Fluorescence Index (FI), which is emission intensity 450/emission intensity 500, at excitation 370 nm, gives an indication of DOC source (allochthonous or autochthonous)

^c^SUVA_254_, which is Abs254/DOC, provides an estimate of the UV-absorbing moieties

^d^Abs_254/365_ gives an indication of molecular weight of DOC molecules

^e^% relative abundance of each DOC component determined via PARAFAC analysis

According to Junk et al. (2011) the black waters of the Rio Negro can vary spatially in terms of their ionic composition, which has an influence on the productivity and abundance of fish in that location. The same is true of dissolved organic carbon (DOC) characteristics. Studies indicate that DOC concentrations in some areas can reach 12 mg L^−1^ (Richey et al. 1990). The characteristics shown in Table 1 indicate that DOC is highly terrigenous, with high aromaticity (high SAC340) and the ability (Ka310) to produce ROS (reactive oxygen species). In fact, Johannsson et al (2017) also analyzed the properties of DOC using absorption and fluorescence indices and parallel factor analysis (PARAFC) of excitation-emission matrices and confirmed those characteristics, and also reported high tryptophan-like fluorescence, relatively high rates of photo-oxidation and ROS levels often higher than the average of what is normally observed for freshwater. These characteristics have direct and diverse effects on fish in this aquatic system, as reported by the same authors for *Hemigrammus levis*.

Photolysis can have a direct effect on DOC, without the need for oxygen which appears at low levels for the typical hypoxic waters of the Rio Negro, to produce dissolved inorganic carbon (DIC, i.e. CO_2_). In the presence of oxygen UV radiation leads to the formation of ROS, degrading DOC, with the possible formation of CO_2_ as well (reviewed by Johannsson et al. 2017). Three direct implications arise from these two processes: (a) the formation of CO_2_ which contributes to water acidification and dispersion into the atmosphere; (b) the formation of H_2_O_2_ which acts on DOC to form small organic molecules; and (c) the formation of H_2_O_2_ which also passes through cell membranes and causes oxidative stress in aquatic organisms (reviewed by Johannsson et al. 2017). Therefore, the presence of DOC in waters such as the Rio Negro and other bodies of water has direct implications, such as those limiting the availability of transition metals, but also undergoing modifications caused by interactions with other environmental factors and negatively affecting aquatic organisms.

Finally, we cannot neglect the effect of climate change on the dynamics of DOC in the waters of the Rio Negro. It can be anticipated that the interaction between DOC and ongoing climate change is complex and multifaceted, involving multiple environmental, biological and chemical processes (Raymond and Saiers 2010, Pagano et al. 2014). The extremes of flood and drought end up redesigning the interaction of the forest with the water bodies of the Rio Negro and, therefore, the quality of the NOM released into the water bodies in the different places along the Rio Negro basin (Marengo et al. 2012; Rodriguez-Zorro et al. 2017). We know that the ongoing changes are making the waters warmer, with less oxygen and more acidic and these three environmental factors have a direct impact on the chemical and physical properties of the DOC.

## Basics of ion transport and inhibition by low pH

**Fig. 2:**
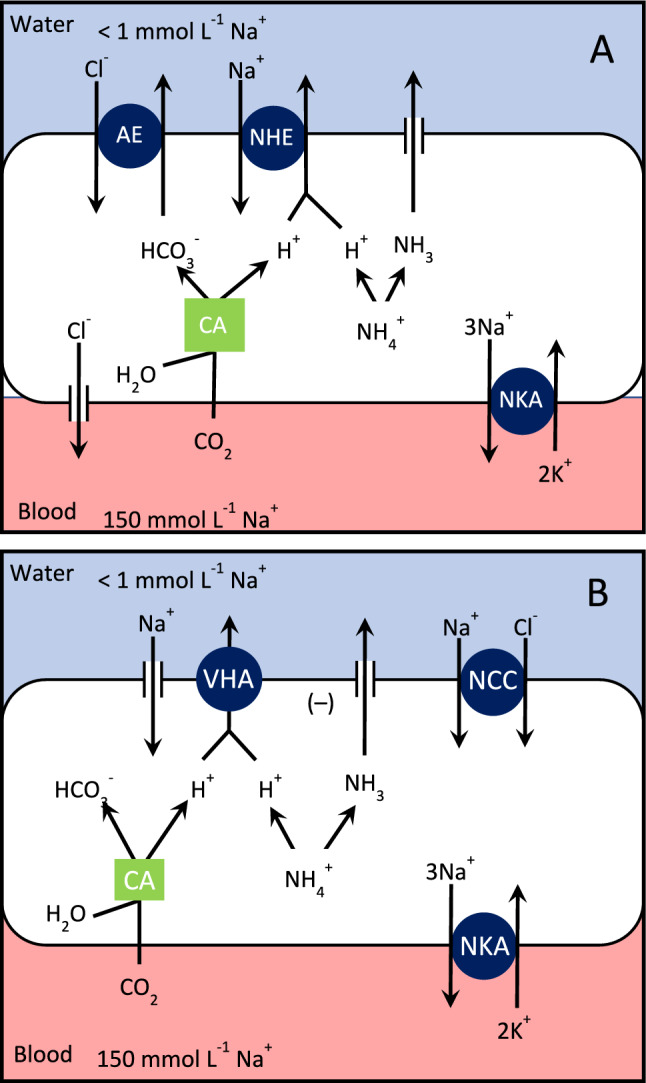
Models of Na^+^ and Cl^−^ transport across gills of freshwater fish. See text for details

Freshwater fish have plasma Na^+^ and Cl^−^ concentrations around 150 and 130 mmol L^−1^, respectively, which are much higher than the surrounding medium (< 1 mmol L^−1^). Consequently, they tend to lose ions by diffusion across the large, permeable gill epithelium. To maintain high internal salt levels, they actively transport salt from the water into the blood across the gills. Our current understanding of the mechanisms involved in ion transport have been addressed in recent reviews (Lee et al. 2022; Zimmer & Perry 2022; Shih et al. 2023), and will only be briefly summarized here. Both Na^+^ and Cl^−^ are moved from the water to the blood across the branchial epithelium in a two-step process. The salts must first be moved from the water into the epithelial cell across the apical membrane, and then from the cell to the blood across the basolateral membrane. There is general agreement that transfer of Na^+^ across the basolateral membrane occurs via the action of Na^+^/K^+^-ATPase (NKA), and that Cl^−^ exits through Cl^−^ channels. Further, pharmacological and immunohistochemical evidence indicates that Cl^−^ is taken up from the water across the apical membrane via a Cl^−^/HCO_3_^−^ exchanger, or anion exchanger (AE; Fig. 2A; Goss et al. 1998; Wilson and Laurent 2002). What is less clear is how Na^+^ enters across the apical membrane.

Currently three different mechanisms for Na^+^ transport have been proposed. The older model for Na^+^ uptake involves an electro-neutral exchange of Na^+^ for H^+^ across the apical membrane by an Na^+^/H^+^ exchanger (NHE; Maetz and Garcia-Romeu 1964; Maetz 1973; Fig. 2A). This mechanism raises significant thermodynamic questions about the gradient driving exchange (Parks et al. 2008). If water pH is equal to or less than intracellular pH, as many freshwaters are, the driving force for exchange must be an inward directed Na^+^ concentration gradient. However, measurements of intracellular Na^+^ (and Cl^−^) levels indicate concentrations around 80 – 90 mmol L^−1^ (Wood and LeMoigne 1991; Morgan et al. 1994), although a recent study suggests a concentration as low as about 5 mmol L^−1^ (Lee et al. 2016). Thus, under usual prevailing conditions it appears the exchanger would act to excrete Na^+^, not take it up. To get around this challenge it has been suggested that H^+^ could be funneled to the exchanger in a metabolon arrangement (Perry and Gilmour 2006). One potential source of H^+^ is provided by the hydration of CO_2_ catalyzed by carbonic anhydrase (it also produces HCO_3_^−^, which can be used for Cl^−^ uptake). An additional source of H^+^ has also been suggested. In this case, NH_4_^+^ is deprotonated to allow NH_3_ to diffuse out through Rh glycoprotein channels in the apical membrane (Wright and Wood 2009).

Because of the challenges for the NHE model, an alternate was proposed (Avella and Bornancin 1989; Lin and Randall 1991, 1993). It couples an apical H^+^-ATPase (VHA), that generates a negative potential across the apical membrane that draws Na^+^ into the cell from the water through an ion-specific channel (Fig. 2B). Evidence for this model includes inhibition of Na^+^ uptake during exposure to the H^+^-ATPase inhibitor bafilomycin in several species of fish (Bury and Wood 1999; Fenwick et al. 1999), as well as immunolocalization of H^+^-ATPase on gill epithelia (Lin et al. 1995; Sullivan et al. 1995; Wilson et al. 2000). However, the inability to identify an epithelial Na^+^ channel has led to the suggestion that acid-sensing ion channels (ASICs) may be involved (Dymowska et al. 2014, 2015).

A third option has recently been proposed in which a Na^+^/Cl^−^ co-transporter moves both salts together across the apical membrane (Fig. 2B). Support for this model comes from immunolocalization on gills of tilapia (*Oreochromis mossambicus*; Hiroi et al. 2008). Still there are questions about how this transporter would operate for Na^+^ and Cl^−^ uptake in freshwater (Hwang 2009).

The first two models for Na^+^ transport differ from the third in that there is no direct linkage between Na^+^ and Cl^−^ uptake. However, there does appear to be the opportunity for an indirect connection between the two salts in the two. If HCO_3_ and H^+^, the counter-ions for transport of Cl^−^ and Na^+^, respectively, are produced in the branchial epithelial cells by the hydration of CO_2_, then it is conceivable that inhibition of Na^+^ uptake would lead to an internal buildup of H^+^ that could limit the production of HCO_3_^−^ for Cl^−^ uptake, and vice versa. Of course, the supply of H^+^ by NH_4_^+^ would limit this linkage.

Exposure to low pH has been shown to inhibit Na^+^ and Cl^−^ uptake in a variety of fish species from North America and Europe (Packer and Dunson 1970; McWilliams and Potts 1978; McDonald and Wood 1981; Gonzalez and Dunson 1987; Wood 1989; Preest et al. 2005). At pH 4.0–4.5 inhibition is complete and irreversible. How low pH is thought to inhibit Na^+^ uptake depends on the mechanism of transport involved. For the first two mechanisms the inhibition is linked to the fact that H^+^ is the ion exchanged, either directly or indirectly, for Na^+^. For Na^+^ transport involving NHE it seems that low water pH (high H^+^ concentration) alters the gradient for exchange such that it strongly favors extrusion of Na^+^ (Parks et al. 2008). It is possible that Na^+^ uptake could occur at low pH via Na^+^/NH_4_^+^ exchange, as suggested by Ito et al. (2014), but the strong inhibition of Na^+^ uptake at low pH seen by Clifford et al. (2022) do not support this. For Na^+^ transport via VHA and a Na^+^ channel, it is thought that low pH increases the rate of H^+^ flux into the cell would make difficult for VHA to generate a sufficient potential to drive Na^+^ uptake (Lin and Randall 1991). Regarding the Na^+^/Cl^−^ co-transporter model it is not clear at all how low pH would inhibit transport. The mechanism of inhibition of Cl uptake is also not clear. Perhaps it occurs through the indirect connection with Na^+^ uptake via carbonic anhydrase. If Na^+^ uptake was reduced at low pH, then this could lead to a buildup of H^+^ in the branchial cell that would hinder further production of HCO_3_^−^ needed for Cl^−^ exchange and thus reduce Cl^−^ uptake (Boisen et al. 2003). However, this would most likely require that both Na^+^ and Cl^−^ transport occur in the same cell types. It is not clear if this is generally the case. In rainbow trout (*Oncorhynchus mykiss*) it appears that Na^+^ and Cl^−^ transport occur in different ionocyte sub-types (Parks et al. 2008). In zebrafish one of the 5 different ionocyte subtypes appears to have both Na^+^ and Cl^−^ transport through a Na^+^/Cl^−^ cotransporter (Bayaa et al.^2009^; Wang et al. 2009).

## Fish with ion transport at low pH

**Fig. 3:**
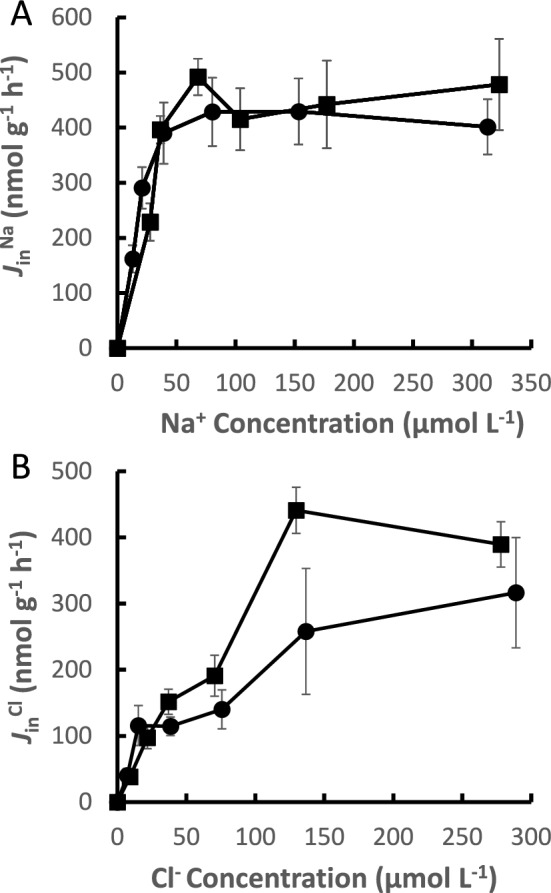
Effect of water Na^+^ concentration (**A**) and Cl.^−^ concentration (**B**) on rate of uptake at pH 6.5 (circles) and 3.5 (squares). Values are means ± SE. Data are from Gonzalez and Preest (1999) and Preest et al. (2005)

Neon tetras were the first species shown to be able to take up Na^+^ at low pH (Gonzalez and Preest 1999). Measurements of uptake upon transfer from pH 6.5 to pH 3.25 did not show even a slight inhibition. It was also shown that Cl^−^ transport in neon tetras was pH insensitive (Preest et al. 2005). In fact, transport kinetics for both Na^+^ and Cl^−^ at pH 3.5 were virtually identical to measurements at pH 6.5 (Fig. 3). In a second study, the congeneric cardinal tetras (*P. axelrodi*) were also found to share the same pH-insensitive transport characteristics with neon tetras (Gonzalez and Wilson 2001). In fact, as more species were surveyed low pH insensitive Na^+^ uptake was found in at least 6 other characids from different genera (Gonzalez et al. 2017, 2018), as well as several species from families outside Characidae, but within the order Characiformes (Gonzalez et al. 2002, 2017, 2021). These findings suggest that the trait may be more widely occurring than originally thought and may even be a general characteristic of the whole order.

In addition to the pH insensitive nature of Na^+^ transport, all the characiform species surveyed share another trait. Kinetic analysis reveals that virtually all K_m_ values are < 60 µmol L^−1^, and most *J*_*max*_ values are ≥ 1000 nmol g^−1^, ^h−1^ (Table 2). Such values indicate an extremely high affinity, high-capacity transport system that is well suited for the extremely ion-poor waters that many of these species inhabit. They insure that even in the most dilute waters rates of Na^+^ uptake will be high.

**Table 2:** Kinetic parameters for Na^+^ uptake in acid-tolerant fish from three families of the order characiformes

Species	K_m_ (µmol L^−1^)	*J*_max_ (nmol g^−1^ h^−1^)	References
Characidae			
Neon tetras	12.9 ± 5.8	448 ± 44	Gonzalez and Preest (1999)
Cardinal tetras	53.7 ± 7.8	773 ± 38	Gonzalez and Wilson (2001)
Black neon tetras	15.7 ± 4.1	1033 ± 59	Gonzalez et al. (2017)
Emperor tetras	41.7 ± 19.8	1342 ± 185	Gonzalez et al. (2017)
Penguin tetras	28.0 ± 6.0	1468 ± 81	Gonzalez et al. (2017)
Rosy tetras	64.1 ± 22.7	1663 ± 201	Gonzalez et al. (2017)
Serpae tetras	34.8 ± 8.7	1111 ± 79	Gonzalez et al. (2017)
Blackskirt tetras	27.7 ± 2.7	691 ± 20	Gonzalez et al. (1997)
*Hemigrammus* sp.	30.9 ± 5.5	1440 ± 76	Gonzalez et al. (2002)
Gasteropelecidae			
Hatchetfish	32.5 ± 6.4	1225 ± 75	Gonzalez et al. (2002)
Alestidae			
Congo tetras	34.9 ± 4.6	1673 ± 66	Gonzalez et al. (2017)

Values are means ± SE

One final unusual characteristic of Na^+^ transport in these fish is that Na^+^ transport kinetics does not appear to acclimate in any way during chronic exposure to water with different Na^+^ levels. Typically, Na^+^ uptake in fish acclimated to waters with higher Na^+^ concentrations have higher K_m_ and lower *J*_max_ values, compared to fish acclimated to waters with low Na^+^ concentrations (McDonald and Rogano 1986; Boisen et al. 2003). This is thought to be a homeostatic response acting to keep rates of Na^+^ uptake constant in the face of changing environmental levels. In contrast, when three species of tetras were held for 1 month in 1 mmol L^−1^ Na^+^ water, there was no change in K_m_ or *J*_max_ values (Gonzalez et al. 2018) relative to fish held in 100 µmol L^−1^ Na^+^ water. The “hard wiring” of these transport parameters has not been seen before.

The Na^+^ transport characteristics, low pH insensitivity, high affinity, and high capacity, appear to be typical of most, if not all characiform fish and when they were first described it was thought that these traits arose as fish colonized and adapted to life in the ion-poor, acidic Rio Negro. Interestingly, however, some of the species examined in this group are not native to the Rio Negro and do not appear to have ever inhabited ion-poor, acidic waters (Gonzalez et al. 2017, 2018). Despite living in less harsh aquatic environments, they still possess the same traits. Further, one species that has been examined, Congo tetras (*Phenacogrammus interruptus*), is native to Africa separating it from South American characiforms for over 100 million years, well before the formation of the Amazon River and Rio Negro (Val and Almeida-Val 1995). Taken together these findings suggest the intriguing possibility that the Na^+^ transport attributes found in characiforms may be ancestral and arose well prior to colonization of the Rio Negro. This may explain why characiforms have been so successful colonizing the Rio Negro. They are the most species-rich group in the river with over 450 different species described so far, which represents 39% of all species in the river (Beltrao et al. 2019).

Interestingly, a second family of fish with species in the Rio Negro, the Cichlidae, were shown to lack any of these transport specializations. Measurement of Na^+^ uptake at low pH showed complete inhibition at pH 4.0, and kinetic analysis generally revealed low affinity, low-capacity transport systems (Gonzalez et al. 2002, 2017, 2021). This may explain why cichlids are less successful colonizing the Rio Negro, making up only about 9% of the fish fauna (Beltrao et al. 2019).

Outside of characiforms only two other species of fish have been shown to possess a Na^+^ transport mechanism that can operate at low pH levels. One species of catfish collected directly from the Rio Negro showed no inhibition of Na^+^ uptake down to pH 3.75 (Gonzalez et al. 2002). In addition, a recent study on zebrafish (*Danio rerio*), a member of the family Cyprinidae, showed that upon exposure to pH 4.0 Na^+^ uptake was initially strongly inhibited, but then recovered back to control levels within 6–8 h (Clifford et al. 2022). A third species is another likely candidate for Na^+^ transport at low pH. Osorezan dace (*Tribolodon hakonensis*) inhabit the acidic (pH 3.4–3.8) Lake Osorezan in Japan (Hirata et al. 1999). Although Na^+^ uptake has not been measured in this fish, its continued survival in such acidic waters raises the possibility.

## Functional studies of ion transport in acidic waters

A series of studies have probed the mechanisms of Na^+^ and Cl^−^ uptake in acidophilic fish species by measuring rates of uptake during exposure to pharmacological agents known to be specific blockers of certain transporters and during exposure to water conditions that could inhibit or stimulate transport if a particular mechanism is employed. This approach can be useful to quickly survey the various mechanisms and possibly rule out alternatives. Looking at Cl^−^ transport first, only one study has attempted to examine the mechanism and the results are consistent with the involvement of Cl^−^/HCO_3_^−^ exchange via an anion exchanger (AE). In that study neon tetras were exposed to the AE inhibitors SITS (4-acetamido-4^′^-isothio- cyanatostilbene-2–2^′^-disulfonic acid) and SCN^−^, and both strongly inhibited Cl^−^ uptake (Preest et al. 2005). Exposure to the carbonic anhydrase inhibitor acetazolamide also strongly inhibited Cl^−^ uptake. Together these findings indicate Cl^−^ uptake in exchange for HCO_3_^−^, which is supplied by the carbonic anhydrase catalyzed hydration of CO_2_. The same study showed that Cl^−^ uptake was unaffected when measured in Na^+^-free water indicating that Na^+^ and Cl^−^ uptake were not directly linked, as with NCC.

In contrast, when we look at Na^+^ transport in characids the picture is anything but conventional. In fact, there is little evidence for roles for NHE or ENaC in Na^+^ transport in this group. In the same study that looked at Cl^−^ transport, neon tetras were also exposed to 4 different amiloride analog inhibitors of NHE; DMA (5-N, N- dimethyl amiloride), MIA (5-N-methyl-N-isopropyl amiloride), and HMA (5-N, N-hexamethylene amiloride) and EIPA (5-N-ethyl-N-isopropyl amiloride) and none had any effect on Na^+^ uptake (Preest et al. 2005). In addition, Na^+^ uptake in tambaqui (*Colossoma macropomum*) a member of the family Serrasalmidae, was also insensitive to DMA (Gonzalez et al. 2021). The findings suggest no role for NHE in Na^+^ uptake. Likewise, neon tetras were insensitive to two ENaC-specific inhibitors, benzamil and phenamil, cardinal tetras were unaffected by phenamil (Wood et al. 2014), and tambaqui were not affected to benzamil (Gonzalez et al. 2021). Cardinal tetras were also unresponsive to amiloride, a general NHE/ENaC inhibitor (Wood et al. 2014).

Consistent with the lack of support for involvement of NHE or ENaC in Na^+^ transport is a more general observation that H^+^ is not exchanged for Na^+^. Exposure to acetazolamide did not inhibit Na^+^ uptake in neon tetras or 4 other tetras in a later study (Gonzalez et al. 2018), although a drop in Na^+^ uptake was seen in cardinal tetras (Wood et al. 2014). A second treatment, high external ammonia (1 mmol L^−1^; HEA) was used to test the role NH_4_^+^ might play in providing H^+^ for Na^+^ uptake. This treatment is designed to limit NH_4_^+^ ability to provide H^+^ for Na^+^ exchange by stopping or reversing diffusive NH_3_ excretion, as proposed by Wood et al. (2014). This treatment failed to inhibit Na^+^ uptake in 6 of 7 different characiforms exposed to it (Wood et al. 2014; Gonzalez et al. 2017, 2018). In a follow up test, two species showed no drop in net ammonia excretion when exposed to Na^+^-free water. Together, these findings suggest that H^+^, whether provided by carbonic anhydrase or NH_4_^+^ are not involved in uptake of Na^+^. Given the complete pH insensitivity these fish display such a conclusion seems reasonable. When tetras are transferred from pH 7.5 to 3.5 this represents a 10,000X increase in H^+^ concentration. If Na^+^ transport relied on H^+^ extrusion, then such a marked increase in external H^+^ concentration would almost certainly impede uptake to some degree, and depending upon the mechanism could even lead to Na^+^ excretion (Parks et al. 2008).

When we look at the final Na^+^ transport option, the Na^+^/Cl^−^ cotransporter (NCC), again we find little support. In neon tetras Na^+^ uptake was unaffected by measurement in Cl^−^ -free water (Preest et al. 2005), and cardinal tetras were insensitive to the NCC inhibitor hydrochlorothiazide (Wood et al. 2014). Taken as a whole, these findings indicate that ion transport is very different in characiforms than other freshwater fishes.

Turning our attention to zebrafish, we see that functional studies again do not support the traditional Na^+^ transport mechanisms in low pH waters. Zebrafish Na^+^ uptake during the first 8 h of exposure to pH 4.0 water was insensitive to the NHE inhibitor EIPA, the ENaC inhibitor phenamil, the ASIC inhibitor DAPI (4′,6-diamidino-2-phenylindole), and the carbonic anhydrase inhibitor acetazolamide (Clifford et al. 2021). Sodium uptake was also unaffected by exposure to Cl^−^-free water or two NCC inhibitors (hydrochlorothiazide or metolazone), indicating no linkage between Na^+^ and Cl^−^ transport. These findings discount the three standard models, not unlike what was seen in the characiforms.

In lieu of the traditional transport mechanisms, Clifford et al. (2021) presented evidence for rapid induction of a novel Na^+^ transporter that involves exchange for K^+^ during exposure to low pH water. Support for a Na^+^/K^+^ exchanger in zebrafish comes from observed changes in the kinetic parameters after 8 h in pH 4.0 water, which indicates fish are switching from the mechanism employed at neutral pH levels to another. Further, after 8 h in pH 4.0 water, raising the water K^+^ concentration above about 10 mmol L^−1^ almost completely inhibited Na^+^ uptake, and transfer to low pH water produced a marked stimulation of net K^+^ loss. These two findings link Na^+^ uptake and K^+^ excretion.

It is theorized that a Na^+^/K^+^ exchanger would be driven by a large outward-directed K^+^ concentration gradient. Due to the activity of Na^+^/K^+^-ATPase, intracellular K^+^ levels typically are at least 80–90 mmol L^−1^ (Wood and LeMoigne 1991; Morgan et al. 1994). Since the outward K^+^ gradient is much larger than the inward Na^+^ gradient outward movement of K^+^ could drive uptake of Na^+^. This would be beneficial in low pH water where the H^+^ gradient is not favorable.

It is worth noting that while both zebrafish and characiform fish both can transport Na^+^ at low pH, there are several significant differences between the two. First, while Na^+^ uptake in zebrafish is initially inhibited at pH 4.0 (Clifford et al. 2022), uptake in tetras is not inhibited at all down to pH 3.25 (Gonzalez and Preest 1999; Gonzalez et al. 2018). In addition, while kinetic parameters for Na^+^ transport in zebrafish change (Clifford et al. 2022) at low pH, they do not in tetras (Gonzalez and Preest 1999). These findings suggest that zebrafish are switching to a new transport mechanism at low pH, while tetras are using the same mechanism in both neutral and low pH waters.

## Molecular and cellular studies of ion transporters

Gene and protein expression studies have been employed extensively to identify ion transporters in many fish species across taxonomic groups and environments. However, few have characterized ion transporter expression in species that are native to acidic waters or quite tolerant of low pH. Expression studies can confirm the presence of ion transporters in the gill epithelium and complement in vivo or in vitro experiments that apply transporter-specific antagonists or environmental manipulations to detect the activity of transporters. Immunolocalization assays enable researchers to visualize the location of ion transporters on ionocyte membranes and document global expression patterns at the gill filament level. Gene expression and manipulation thereof with direct measurement of transport at the organ level (*e.g.* SIET, Na green) are newer approaches that provide a more refined view of ion transport. Below, we focus on presenting data from studies that employed these approaches to identify key ion transporters in species native to acidic waters or those that show exceptional tolerance to low pH as noted in earlier sections.

### ATPases

P-type Na^+^/K^+^ ATPase (NKA) and V-type H^+^ ATPase (VHA) are considered to be the two likeliest candidates that establish a favorable electrogenic gradient for Na^+^ uptake in freshwater teleost. Both ATPases have been detected in the gill epithelium of species native to acidic waters but display species-specific expression patterns during low pH exposure. The Rio Negro resident tambaqui (*Colossoma macropomum*), which, as described above, possesses a pH-insensitive in vivo Na^+^ uptake rate, expresses VHA and NKA proteins on the apical and basolateral membrane, respectively, of gill ionocytes. Upon transfer from pH 6.5 to 3.5, tambaqui show no change in gill morphology, ionocyte location, protein density or activity of both ATPases (Gonzalez et al. 2021). Different patterns have been reported for Osorezan dace (*Tribolodon hakonesis*) that lives in a volcanic lake with a pH of 3.5. Hirata et al. (2003) performed immunolocalization assays to reveal high expression of NKA protein on the basolateral membranes of chloride cells (equivalent to ionocytes) in the gills whereas Western blot analyses revealed low expression of VHA that did not vary greatly when dace were transferred to pH 3.5. Another departure noted was the dramatic gill remodeling during low pH exposure with chloride cells forming clusters around apical crypts and increasing the percent chloride cell area (Kaneko et al. 1999). One could presume that these changes in dace gills led to an increase in NKA density and activity in order maintain or upregulate Na^+^ uptake rates as plasma Na^+^ levels remain high following transfer to low pH (Hirata et al. 2003). However, rate of Na^+^ transport has not been measured. Mozambique tilapia (*Oreochromis mossambicus*) are not native to acidic waters but are euryhaline fish capable of tolerating low pH freshwater. Furukawa et al. (2011) reported that changes in tilapia gills exposed to pH 4 water (and low Na^+^ levels) were similar to that described above in dace (Kaneko 1999), with larger ionocytes, forming a greater number of apical crypt complexes, expressing NKA protein on basolateral membranes and plasma Na^+^ levels not differing from neutral pH values. It is interesting to note that this morphological remodeling noted in both dace and tilapia may reflect their diadromous natural history as it is reminiscent of the pattern of multiple chloride cells/apical crypt formations described in gills of seawater teleosts (Karnaky 1986). It has been proposed that this remodeling could provide a more protective microenvironment for Na^+^ uptake, such as enhanced buffering of acidic water within the crypt (Furukawa et al. 2011).

In contrast to the dace and tilapia, zebrafish, a true freshwater species that is not native to acidic habitats but tolerates low pH (3.5–4.0, Kumai et al. 2011) appears to rely upon VHA as the primary ATPase to power the significant upregulation of Na^+^ uptake that occurs over several days (Shir-Mohammadi and Perry 2020) to counteract the Na^+^ efflux stimulation induced by low pH exposure (Kumai et al 2011). This response avoids a large drop in whole-body Na^+^ levels (Kumai et al. 2011) and has been well characterized in both zebrafish adult gill and larval yolk sac epithelia using a combination of molecular analyses and manipulation (*i.e*. transporter gene knockdown), in situ protein expression and in vivo SIET (scanning ion selective electrode technology) the latter used to directly measure ion fluxes at the surface of yolk sac epithelium (see reviews by Kwong et al. 2014; Shih et al. 2023). Apical VHA is highly expressed only in one of the five types of ionocytes, the HR (VHA-rich) cells that are considered to be the primary site of Na^+^ uptake and acid secretion. Support for this assertion comes from several studies that report increases in HR ionocyte size and density, VHA mRNA expression, H^+^ efflux and Na^+^ influx (Yan et al. 2007; Chang et al. 2009; Horng et al. 2009; Kumai and Perry 2011; Shih et al. 2022) following several days at pH 4.0. Additionally, Horng et al. (2009) revealed that the surface area of an “alveolar-type” apical opening that localizes to HR cells increases in in zebrafish embryos held at low pH. These apical coverings are reminiscent of the apical crypts noted above in euryhaline dace and tilapia held at low pH and suggest a structural means to provide a protective microenvironment to enhance Na^+^ uptake. Knockdown of an A subunit of VHA resulted in reduced H^+^ efflux at the HR surface, whole-body Na^+^ levels and survival rate in low pH (Horng et al. 2007). The four other ionocytes do not express VHA but all five ionocytes express different isoforms of NKA on the basolateral membrane, with NCC (Na-Cl cotransport) cells considered a secondary route for Na^+^ uptake. Studies so far have not reported changes in NKA activity or expression with low pH treatment (Guh et al. 2015; Shir-Mohammadi and Perry 2020).

Recent transcriptomic studies of fish that inhabit all three water chemistries of the Amazon basin—blackwater, clearwater and whitewater—have reported different patterns of ATPase gene expression when exposed to these media. Both the characiform sardine (*Triportheus albus*) and the yellow peacock bass (*Cichla ocellaris monoculus*), a cichlid, showed a blackwater-biased expression of both NKA and VHA subunits or paralogs when held at pH 4.6 and 4.2 respectively (Araújo et al. 2017; Willis et al. 2022).

### Apical Na^+^ transporters–exchangers and channels

The means by which Na^+^ is transported across the apical membrane of freshwater fish gill ionocytes appears to involve a few candidate transporter classes—electroneutral exchangers, cotransporters and Na^+^ channels. Gene expression studies have identified each of these classes of transporters in a few species that are native to or tolerant to acidic pHs. The Osorezan dace (*T. hakonesis*), shows increased transcript levels for Na^+^/H^+^ exchanger, isoform 3 (NHE3) when transferred and held at pH 3.5 for several days (Hirata et al. 2003). Species that can reside in the acidic blackwater of the Amazon region showed significantly higher gene expression of NHE6 (*T. albus*) and NHE2 (*C. ocellaris monoculus*), when held in acidic blackwater (pH 4.2–4.6) with the cichlid species also showing a slight (but not significant) increase in expression of NHE3, NCC (Na^+^ Cl^−^ cotransporter) and an acid-sensing ion channel ASIC2, a member of the superfamily of epithelial Na^+^ channels (eNaC) (Araújo et al. 2017; Willis et al. 2022).

Comprehensive studies that examined and/or manipulated gene/protein expression of transporters and employed SIET or other techniques (Sodium Green fluorescent probe) to quantify and localize ion fluxes, have been able to confirm the presence and function of one or more of these classes of Na^+^ transporters in the gill of species that tolerate low pH treatment. Both NHE3 and NCC gene expression increases in the Mozambique tilapia (*O. mossambicus*) when transferred to pH 4.0. At the protein level, NHE3 was localized to the apical membrane of NKA-rich ionocytes that were larger and in greater number in tilapia held at low pH. Interestingly, it was the NHE3 cells that congregated into clusters with apical crypts in low pH gills. This contrasts with NCC ionocytes (also NKA-rich) that were also greater in density (but not size) but remained isolated (Furukawa et al. 2011).

The vast body of work on zebrafish has yielded results suggesting multiple avenues for Na^+^ uptake. Although both NHE3b and ASIC4.2 are expressed in these VHA-rich ionocytes (Dymowska et al. 2015; Shir-Mohammadi and Perry 2020), only Shir-Mohammadi and Perry 2020 examined expression patterns during long term (4 days) pH 4.0 exposure and found that zebrafish embryos showed greater HR cell-specific expression of NHE3. Dymowska et al. (2015), reported ASIC gene expression and function in adult gills that were acclimated (7 days) to ultra-low Na^+^ water and noted that this treatment involved a pH drop from 7.0 to 6.0 representing a fourfold proton concentration increase. Concurrently, Rhesus (Rh) glycoprotein gene, rhcgb, that encodes for an ammonia (NH_3_) facilitated diffusion transporter, and carbonic anhydrase, that generates protons from CO_2_ hydration, are both expressed to higher levels in HR cells from zebrafish reared at low pH (Shir-Mohammadi and Perry 2020). Together, these findings point to an HR-cell metabolon, an integrated system where ammonia excretion, which is elevated at low pH, drives the greater rate of Na^+^ uptake (Kumai and Perry 2011). Ammonia (NH_3_) diffuses outward through Rhcg and is immediately protonated by external protons. In the HR cell, protons from dissociated NH_4_^+^ or CA production diffuse down the H^+^ gradient through NHE3 in exchange for Na^+^ moving into the cell. The elevated VHA gene expression noted in HR-cells during low pH holding is thought to increase acid excretion rate to maintain this ammonia trapping mechanism rather than an inward electrogenic gradient for Na^+^ entry through ASIC (Shir-Mohammadi and Perry 2020). This ammonia-driven Na^+^ uptake mechanism via Rhcg/NHE3 at low pH was echoed in the medaka (*Oryzias latipes*) at pH 6 exposure (Wu et al. 2010) and hinted at in the blackwater-biased gene expression of both NHE and rhcg genes in Amazonian *T. albus* and *C. ocellaris monoculus* (Araújo et al. 2017; Willis et al. 2022). However, questions remain as to how both NHE and VHA could operate concurrently given that proton pumping of VHA would abolish the proton gradient necessary for NHE3 function.

A recent newcomer, NCKX, a K^+^—dependent Na^+^/Ca^2+^ exchanger, was found to have six isoforms expressed in gill tissue of zebrafish and characterized through in vivo studies (see above) to play a role in maintaining Na^+^ uptake during low pH exposure. This study raises questions regarding the role of other Na^+^ exchangers and channels that have been identified in zebrafish and other acid-tolerant species (Clifford et al. 2021). As has been stated in numerous studies, expression and function of these transporters may follow a species-specific pattern.

## Future directions

We now know that the ability to transport ions in extremely acidic waters is more common in fish than was first thought, but exactly how common is not clear. Certainly, in characiform fishes it appears to be the rule rather than the exception. However, barely a dozen species out of over 2000 (from 6 of 20 families) in the Order have been examined so it is not well established how prevalent the trait really is. Outside of the Characiformes, only a few isolated species have been shown to possess ion transport that functions at low pH, a Rio Negro catfish (Gonzalez et al. 2002), North American yellow perch (Freday and McDonald 1988), and Asian zebrafish (Clifford et al. 2021). In these cases, it has yet to be determined if these are lone instances or reflect a trait shared among other species in their respective groups. Given how few species have been examined overall, it seems likely that there are other species out there with this ability. It seems reasonable that the place to look for acid-tolerant species are naturally occurring acid habitats, and these are found all over the world. A survey of species from these waters would almost certainly be rewarded.

We still have much to learn about the mechanism(s) of Na^+^ transport employed at low pH by acid tolerant species. Evidence so far seems to suggest that characiform fish do not utilize versions of the traditional Na^+^ transport mechanisms, nor do they appear to use a Na^+^/K^+^ exchanger as seen in zebrafish (although it cannot be completely ruled out yet). What is the mechanism? To answer that question, we will need to combine functional assays of transport with cellular and molecular techniques to identify the arrangement of transport components involved. Traditional in vivo ion flux measurements, either whole-body or SIET measurements at the at the epithelial surface, with manipulation of water chemistry or use of pharmaceutical antagonists of ion transporters are valuable for characterizing the kinetics of ion transport and potentially narrowing down the list of possible ion transporter candidates. However, we must be mindful that species- and isoform-specific affinity differences exists for several of the inhibitors used to probe ion transporter function (e.g. amiloride analogs and NHE isoforms—Ishizuka et al. 2019, Schwark et al. 1998, eNaCs, ASICS—Dymowska et al 2015, Garvin et al. 1985). But confidence can be built by combining these in vivo approaches with gene and protein expression analyses or manipulation thereof. This strategy has been successfully employed in the development of the zebrafish model of Na^+^ uptake in long term exposure to low pH water (Kwong et al. 2014), a species that is sensitive but tolerant to low pH. Gonzalez et al (2021) is the first study to take this integrated approach to examine the pH-insensitive native species, tambaqui. Far more work needs to be done to determine if the acid insensitivity of Na^+^ uptake in characid species is a consequence of a hard-wired gene/protein expression and gill/ionocyte morphology and function that maintains Na^+^ transport or are there an array of dynamic responses, acute and long-term, resulting in changes in transporter classes or isoforms.
